# Circulating MicroRNAs as Biomarkers for Sepsis

**DOI:** 10.3390/ijms17010078

**Published:** 2016-01-09

**Authors:** Fabian Benz, Sanchari Roy, Christian Trautwein, Christoph Roderburg, Tom Luedde

**Affiliations:** Department of Medicine III, University Hospital RWTH Aachen, Pauwelsstrasse 30, Aachen 52074, Germany; fab-benz@web.de (F.B.); sroy@ukaachen.de (S.R.); ctrautwein@ukaachen.de (C.T.)

**Keywords:** miRNA, biomarker, critical illness, sepsis

## Abstract

Sepsis represents a major cause of lethality during intensive care unit (ICU) treatment. Pharmacological treatment strategies for sepsis are still limited and mainly based on the early initiation of antibiotic and supportive treatment. In this context, numerous clinical and serum based markers have been evaluated for the diagnosis, the severity, and the etiology of sepsis. However until now, few of these factors could be translated into clinical use. MicroRNAs (miRNAs) do not encode for proteins but regulate gene expression by inhibiting the translation or transcription of their target mRNAs. Recently it was demonstrated that miRNAs are released into the circulation and that the spectrum of circulating miRNAs might be altered during various pathologic conditions, such as inflammation, infection, and sepsis. By using array- and single PCR-based methods, a variety of deregulated miRNAs, including miR-25, miR-133a, miR-146, miR-150, and miR-223, were described in the context of sepsis. Some of the miRNAs correlated with the disease stage, as well as patients’ short and long term prognosis. Here, we summarize the current findings on the role of circulating miRNAs in the diagnosis and staging of sepsis in critically ill patients. We compare data from patients with findings from animal models and, finally, highlight the challenges and drawbacks that currently prevent the use of circulating miRNAs as biomarkers in clinical routine.

## 1. Introduction

Sepsis and septic shock syndrome represent major issues in today’s medicine. In developed countries the incidence is estimated to be up to 100 cases per 100,000 population [1], with approximately 2% of all hospital patients presenting with sepsis at hospital admission [2,3]. Despite great progress in intensive care treatment, including implementation of evidence-based guidelines, it is still associated with an important mortality: between one-fifth to half of all sepsis patients do not survive and die mostly due to multiorgan failure [4,5].

Current guidelines for the treatment of sepsis recommend a timely and appropriate antibiotic therapy, accompanied by fluid resuscitation, use of vasopressors, if needed, and a supportive therapy for organ failure [5]. The mortality of patients with sepsis could be further reduced by introducing early goal-directed therapy with early hemodynamic stabilization of the patient within the first 6 h [6]. In line, delayed administration of appropriate antibiotic treatment was associated with an increased mortality in patients with sepsis [7]. Despite these efforts, mortality of sepsis has remained high [3,4], underlining a need for a better stratification of critically ill and septic patients to identify those with increased risk of death. In this context, laboratory biomarkers and clinical scores for early diagnosis of septic disease might be of exceptional clinical significance. However the specificity and sensitivity of current biomarkers, including C-reactive protein (CRP), Procalcitonin (PCT), and Interleukin-6 (IL-6) are limited in this setting [8,9], and recent studies have suggested the use of new biomarkers for sepsis, including analysis of circulating miRNAs [10,11]. In the present review we summarize current findings on a potential role of circulating miRNAs in the diagnosis and staging of sepsis and will focus on current challenges and drawbacks preventing the use of circulating miRNAs as biomarkers in clinical routine.

## 2. Biomarkers in Sepsis

The National Institutes of Health defines a biomarker as a “characteristic that can be objectively measured and evaluated as an indicator of normal biological processes, pathological processes, or pharmacological responses to a therapeutic intervention” [12]. Sepsis can be defined as the (suspected) presence of infection (invasion of microorganisms in the bloodstream) in combination with systemic manifestations of inflammation known as systemic inflammatory response syndrome (SIRS) [5]. Goals of analyzing biomarkers in the setting of sepsis are, therefore, the determination of an infective condition and, consequently, the distinction between SIRS and sepsis, on the one hand, and the prediction of patients prognosis during therapy on the other [2]. Microbiological culture still represents the gold standard in distinguishing sepsis from other non-infectious diseases [2,5]; however, this technique is time-consuming and often related to false negative results. Therefore, other parameters, such as CRP and PCT, are measured in addition to blood cultures when sepsis is suspected. CRP is an acute phase protein and is released from the liver after stimulation by IL-6 [13], underlining that CRP is not specific for infections. It can also be elevated after burns and cannot differentiate between infected and non-infected burn patients [14]. However, the level of CRP serum levels correlate with the severity of infection [15] and a decrease in CRP level after antimicrobial therapy in sepsis patients correlates with the effectiveness of treatment [16]. A recent meta-analysis showed only a low sensitivity and specificity for CRP (0.75 and 0.67) to indicate an infection.

Due to this limited diagnostic value, CRP is not recommended in the current sepsis guidelines as a sepsis biomarker [5]. In contrast, elevated serum PCT levels are more closely associated with sepsis and are, therefore, listed by the Sepsis Guidelines as a diagnostic biomarker for infection and septic disease [5]. PCT is a prohormone of calcitonin and is secreted in healthy individuals only in neuroendocrine cells of the thyroid gland. During an infection, however, PCT is released from almost all tissues and cells [17]. PCT is superior to CRP in the diagnosis of bacterial infections [18], and a recent meta-analysis showed that PCT might be useful to guide antibiotic therapy in septic patients [19]. In addition to CRP and PCT, the proinflammatory cytokine IL-6 is often measured in the context of sepsis. IL-6 is secreted by innate immune cells and acts as an activator of the acute phase proteins. IL-6 is elevated in the serum of sepsis patients, and IL-6 levels correlate with the severity of septic shock. In line, IL-6 levels could be established as a prognostic marker in sepsis-patients [20,21,22]. However, IL-6 also increases in non-infectious diseases, such as trauma, surgery, and stroke [23,24]. Other biomarkers, such as soluble urokinase-type plasminogen activator receptor (suPAR), and CD64, are still experimental and their diagnostic and prognostic values require validation in larger cohorts [8].

Given the limitations of established markers, tremendous efforts have been initiated to identify novel markers in the context of septic diseases. Recently, more than 180 different potential biomarkers have been examined for their potential to detect inflammation, infection and sepsis in numerous clinical and pre-clinical studies [8,9,25]. However, until today, no single biomarker with sufficient sensitivity and specificity could be identified [9,26]. Therefore, novel biomarker classes, such as circulating miRNAs might offer new perspectives and recently received considerable attention in the field of sepsis research.

## 3. Biogenesis and Release of miRNAs

**Figure 1:**
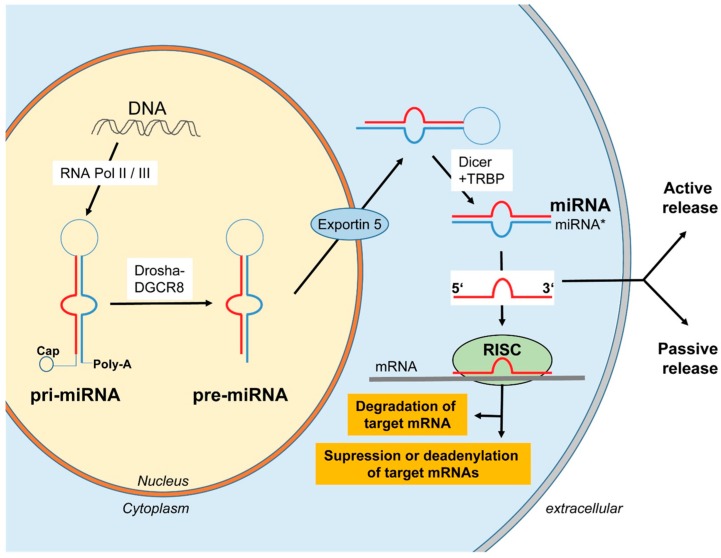
Biogenesis of miRNAs.

MicroRNAs (miRNAs) represent a novel group of small (20–24 nucleotides) RNA molecules that do not encode for proteins, but regulate gene expression. In 1993, miRNAs were first described in nematodes by the groups of Victor Ambros und Gary Ruvkun [27,28]. In further studies, the principle of miRNA regulation could be expanded to other organisms and, to date, more than 1800 miRNAs have been described in humans [29]. miRNAs are transcribed by RNA polymerase II (more rarely by RNA polymerase III), resulting in a primary miRNA (pri-miRNA) transcript with a length of 500–3000 nucleotides, characterized by a loop and containing a typical poly-A tail at the 3′ end and a 7-methylguanosine-Cap at the 5′ end. The primary miRNAs is cleaved into a 70–80 nucleotide precursor miRNA (pre-miRNA) by the “microprocessor complex” which consists of the ribonuclease III enzyme Drosha and the dsRNA-binding protein DGCR8. This pre-miRNA is actively exported from the nucleus into the cytoplasm through the nuclear export receptor exportin-5. In the cytoplasm, the pre-miRNA is processed by the RNase III endonuclease Dicer protein in a 17–24 nucleotides long ds-miRNA. This so-called “miRNA/miRNA * duplex” is unwound and single-stranded, whereas the miRNA * strand is usually degraded. After binding to the Argonaute protein the miRNA strand is integrated into the “RNA-induced silencing complex” (RISC) where they bind to partial or full-complementary sequences in the 3′ or 5′ untranslated region (UTR) of the target mRNAs. Of note, partial complementary binding leads to a translational repression, while complete complementary binding leads to the degradation of the target mRNA (Figure 1, [30,31,32]).

It is estimated that the miRNA genes account for only about 1% of the human genome, but regulate up to 60% of all protein-coding genes [33,34]. miRNAs inhibit the expression of their target genes by base pairing to the 3′ UTR of their mRNA. Thus, one miRNA can affect the transcription of several hundred mRNAs. Therefore miRNAs are suggested to be part of complex regulatory networks in the gene expression of both physiological and pathophysiological processes. Aberrant miRNA expression were described in highly-regulated mechanisms such as development, aging, and cell death [35], but also in the initiation of complex diseases such as infection, inflammation and sepsis [36,37,38,39].

miRNAs have also been detected in the blood and might serve as biomarkers [36,40,41,42]. Circulating miRNAs are extraordinarily stable in conditions that usually would degrade most RNAs, e.g., high or low temperature, changes in pH, and repetitive freezing and thawing cycles. Protection of circulating miRNA results from their association to different RNA-binding proteins and lipoprotein complexes or inclusion into microparticles. It was recently suggested that these exosomes might transfer miRNAs to modulate biological activities in recipient cells [43,44], suggesting that alterations in the release of miRNAs might represent a mechanism of intercellular communication. In addition to their stability, circulating miRNAs harbor several advantages compared to “conventional” protein based markers: miRNAs are relatively small, do not undergo post-processing modifications, and have a less complex chemical structure. Thus, many authors hypothesized that circulating miRNAs might be superior to other classes of serum based biomarkers [45].

## 4. miRNAs in the Pathophysiology of Sepsis

**Figure 2:**
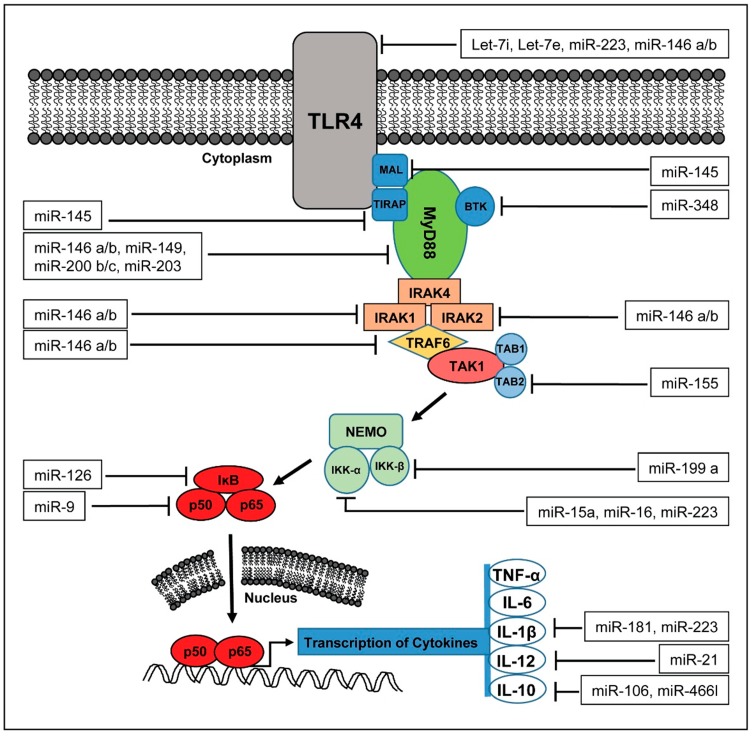
Various miRNAs interact with the TLR4 pathway.

**Figure 3:**
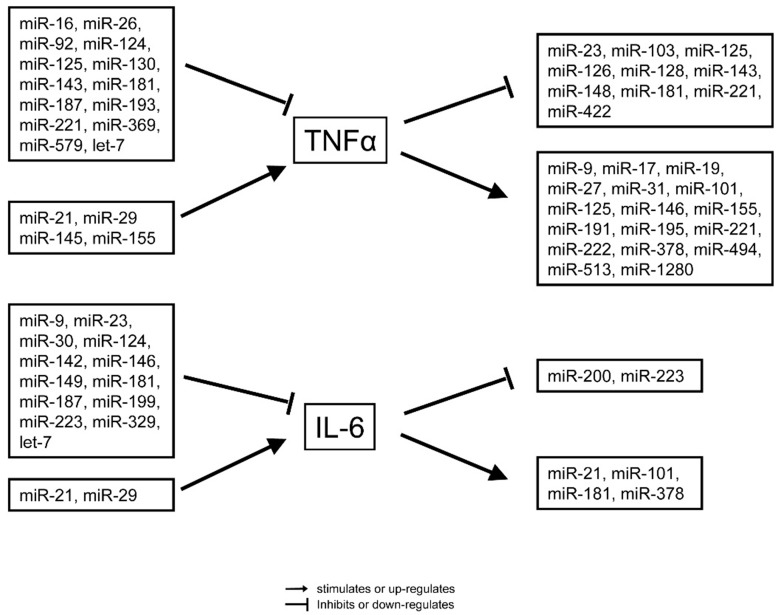
Modulation of the pro-inflammatory cytokines TNF-α and IL-6 by various miRNAs and *vice versa*.

miRNAs are critically involved in the innate and adaptive immunity in pathological disorders including atherosclerosis, rheumatoid arthritis, diabetes, and bacterial infection [46]. It was suggested that various miRNAs directly target the tumor necrosis factor (TNF) pathway, a major regulator of pro-inflammatory processes in sepsis. Increased expression of miR-155 was observed in macrophages and liver tissue upon stimulation with lipopolysaccharides (LPS)/TNF. Moreover, it was demonstrated that miR-155 transgenic mice display more severe disease after treatment with TNF [47]. Moreover, injection of miR-511 into mice reduced TNF receptor-1 (TNFR1) expression levels and thereby protected against TNF dependent endotoxic shock syndrome [48]. Apart from TNF, miRNAs were shown to play a functional role in the regulation of sepsis by targeting the toll-like receptor (TLR)/NF-κB signalling pathways. TLRs are part of the innate immune system mediating systemic inflammatory responses to pathogens during sepsis [49]. TLRs are expressed on macrophages, dendritic cells, and various non-professional antigen-presenting cells and recognize pathogen-associated molecular patterns (PAMPs), which are expressed by microbial pathogens, or danger-associated molecular patterns (DAMPs) released from necrotic or dying cells [50,51,52]. Currently 10 human and 12 murine TLRs have been described [50]. TLR4 is activated by bacterial lipopolysaccharide (LPS) and is, therefore, suggested to be most relevant in sepsis [53]. Various miRNAs are regulated by TLR4 signalling in innate immune cells. On the other hand, it was shown that several mRNAs encoding for components of the TLR signalling system are targeted by these miRNAs (Figure 2). Moon *et al.* [54] showed that miR-15a/16 deficiency led to augmented phagocytosis with an increase in TLR4 expression by targeting its transcriptional regulator PU.1. Another study revealed miR-146a to be a dual transcriptional regulator of TLR4 in the human monocytic cell line THP-1 sepsis cell model [55]. In this study, miR-146a not only promoted binding of the transcriptionnal repressor RelB to the TNF-α promoter, but also enhanced the interactions between the Ago2 and RBM4 members of the translational repressor (miRISC) complex. In addition, miR-30a was predicted to regulate STAT1 (signal transducer and activator of transcription 1) and MD-2 (=Lymphocyte antigen 96) expression in monocytes [56]. The hyper-inflammatory stage during sepsis is associated with enhanced expression of adhesion molecules on monocytes, neutrophils and endothelial cells [57]. Finally, miR-23b was reported to play an important role in progression of sepsis by inhibiting the expression of NF-κB, TNF-α, IL-6, ICAM-1, E-selectin, and VCAM-1. In this scenario, miRNA at the same time regulate the expression of sepsis related genes, such as IL-6 and TNF, and are regulated in their own expression by these factors (Figure 3), highlighting the deep integration of miRNAs in the pathophysiology of septic disease.

## 5. Circulating miRNAs as Biomarkers for Sepsis

As pointed out, miRNAs have been detected in different body fluids, including blood, sweat, and urine. In the last years, whole panels of deregulated miRNAs have been described in the blood of patients with inflammatory/infectious diseases, suggesting that circulating miRNAs might also be suitable as biomarkers in the setting of critical illness and sepsis (Table 1).

**Table 1:** Overview of studies that examined circulating miRNAs as biomarkers in critically ill patients with or without sepsis.

Reference	Year	Study Population	Analyzed miRNAs	Results
[58]	2009	24 Sepsis, 32 healthy controls	miR-150,	↓ (in Sepsis)
			miR-182,	↑
			miR-342-5p,	↓
			miR-486	↑
[59]	2010	50 Sepsis, 30 SIRS, 20 healthy controls	miR-146a,	↓ (and Sepsis *vs.* SIRS)
			miR-223,	↓ (and Sepsis *vs.* SIRS)
			miR-126,	↓
			miR-132,	↔
			miR-155,	↔
			let-7i	↔
[60]	2012	12 Sepsis-Survivor und 12 Sepsis-Non-Survivor for Microarray-Scan, 66 Sepsis-Survivor und 52 Sepsis-Non-Survivor for Validation	miR-574-5p,	↑ (in Survivor)
			miR-297	↓ (in Survivor)
[61]	2012	117 Sepsis-Survivor, 97 Sepsis-Non-Survivor	miR-223,	↓ (in Non-Survivor),
			miR-16,	↓
			miR-15a,	↑
			miR-15b,	↔
			miR-122,	↑
			miR-193b *,	↑
			miR-483-5p,	↑
			miR-451,	↔
			miR-486-5p,	↔
			miR-378,	↔
			miR-499-5p,	↔
			miR-206	↔
[62]	2012	166 Sepsis, 32 SIRS, 24 healthy controls	miR-15a,	↑ (and Sepsis < SIRS)
			miR-16	↑ (and Sepsis = SIRS)
[63]	2012	123 severe Sepsis, 43 mild Sepsis, 24 healthy controls	miR-223,	↑ (mild > severe Sepsis)
			miR-15b,	↑ (mild > severe)
			miR-483-5p,	↑ (mild > severe)
			miR-499-5p,	↓ (severe < mild)
			miR-122,	↓ (severe = mild)
			miR-193b *	↓ (severe = mild)
[64]	2012	17 ICU patients without Sepsis, 36 with Sepsis	miR-181b	↓ (in Sepsis)
[11]	2013	138 ICU with Sepsis, 85 ICU without Sepsis, 76 healthy controls	miR-150	↓
[65]	2013	14 SIRS, 14 Sepsis	miR-146a	↓ (in Sepsis)
[66]	2013	22 Sepsis, 22 SIRS, 17 healthy controls	miR-342-3p,	↓ (Sepsis < SIRS)
			miR-3173-5p,	↓ (Sepsis < SIRS)
			miR-191 iso,	↓ (SIRS = Kontrolle)
			miR-150,	↓ (Sepsis < SIRS)
			miR-4772-3p,	↑ (Sepsis = SIRS)
			miR-4772-5p iso,	↑ (Sepsis > SIRS)
			miR-4772-5p	↑ (Sepsis = SIRS)
[67]	2013	3 Sepsis-Survivor, 3 Sepsis-Non-Survivor, 3 ICU-Nonsepsis	miR-466I	↑
[10]	2014	138 ICU with Sepsis, 85 ICU without Sepsis, 76 healthy controls	miR-133a	↑
[68]	2014	223 ICU-Patienten (138 Sepsis, 85 Non-Sepsis), 76 healthy controls	miR-122	
[69]	2014	232 Sepsis (106 Non-Survivor = Non-S, 126 Survivor = S), 24 healthy controls	miR-122,	↑ (Sepsis > C, Non-S > S)
			miR-193b *,	↑ (Sepsis = C, Non-S > S)
			miR-483-5p,	↑ (Sepsis > C, Non-S > S)
			miR-574-5p	↑ (Sepsis > C, Non-S = S)
[70]	2014	123 Sepsis (54 with coagulation disorders) (=CA) *vs.* 69 without coagulation disorders) (=CN)	miR-122,	↑ (in CA)
			miR-223,	↔
			miR-15a,	↔
			miR-16,	↔
			miR-193b *,	↔
			miR-483-5p	↔
[71]	2014	40 children with sepsis, 20 SIRS, 15 healthy controls	miR-21,	↔
			miR-125b,	↔
			miR-132,	↔
			miR-146a,	↑
			miR-155,	↔
			miR-223	↑
[72]	2015	137 Sepsis, 84 Non-Sepsis, 75 healthy controls	miR-223	↔
[73]	2015	40 septic shock, 29 Sepsis, 24 healthy controls	miR-150,	↑ (Sepsis = controls)
			miR-146a,	↔
			miR-223	↔
[74]	2015	22 Urosepsis, 20 healthy controls	let-7a,	↓
			miR-150,	↓
			miR-1249,	↔
			miR-199b-5p	↔
[75]	2015	46 with Neonatal-Sepsis, 41 with Neonatal-Pneumonie as controls	miR-15a,	↑ (in Sepsis)
			miR-15b,	↔
			miR-16,	↑
			miR-206,	↔
			miR-223,	↔
			miR-378,	↔
			miR-451	↔
[76]	2015	70 Sepsis, 30 SIRS	miR-21,	↔
			miR-25,	↓ (in Sepsis)
			miR-203,	↔
			miR-423-5p,	↔
			miR-513a-5p,	↔
			miR-503	↔

SIRS, systemic inflammatory response syndrome; ↓, downregulated; ↑, upregulated; ↔ unchanged.

### 5.1. miR-25

To identify alterations in circulating microRNAs between SIRS and sepsis, Yao and colleagues used the liquid bead array method on samples from five critically ill patients with and without proven bacterial infection [76]. After combining three different statistical approaches, six miRNAs were selected for further confirmation in a well characterized cohort of 70 patients with sepsis and 30 patients with non-infectious SIRS. Of the six selected miRNAs (miR-21, miR-25, miR-203, miR-423-5p, miR-503, miR-513a-5p), only changes in miR-25 levels remained significant in the larger cohort. Notably, miR-25 displayed a superior diagnostic accuracy for sepsis compared to well established markers such as CRP and PCT according to ROC curve analysis. Moreover, low miR-25 levels were associated with impaired patients’ prognosis, suggesting that miR-25 can be used as a biomarker for the diagnosis and assessment of sepsis. Notably, a dysregulation of circulating miR-25 was also described in other inflammatory settings such as acute aerobic exercise and ozone exposure [77,78], further underlining the role of miR-25 in the context of inflammation.

### 5.2. miR-122

miR-122 represents a liver cell specific miRNA. Elevated concentrations of miR-122 were described in the context of liver injury and liver cell death [68,79,80]. Moreover alterations in miR-122 levels were described in the context of chronic liver diseases and hepatocellular carcinoma [81,82]. Wang and colleagues first described a potential role of circulating miR-122 in critical illness and sepsis: by using Solexa sequencing followed by PCR in samples of 214 patients with sepsis (117 survivors and 97 non-survivors), miR-122 was identified as part of a 6 miRNA signature (miR-223, miR-15a, miR-16, miR-122, miR-193 *, and miR-483-5p) that predicted patients’ short and long term survival with high accuracy [69,70]. Moreover, the authors suggested that alterations in miR-122 levels might also be used as diagnostic biomarker for sepsis. However, it was shown later that elevated miR-122 in critically ill patients primarily correlated with the presence of liver injury, because in a large cohort of critically ill patients only those with elevated aspartate aminotransferase (AST)/alanine aminotransferase (ALT) levels also displayed elevated miR-122 levels [68]. Moreover, elevated miR-122 levels were found in patients with elevated activated partial thromboplastin time (APTT) ratios and low fibrinogen/antithrombin III as a surrogate for aberrant liver function. Finally, it was demonstrated that elevated miR-122 levels indicate a poor neurological outcome in patients after cardiac arrest, suggesting that organ malperfusion is related to the up-regulation of circulating miR-122. Altogether, circulating miR-122 represents a highly specific marker for liver injury in the setting of critical illness and sepsis rather than a specific marker for bacterial infection or sepsis *per* *se*. This hypothesis is further corroborated by similar findings from experimental sepsis models, as well as other diseases models (hepatitis-C infection, liver fibrosis), where miR-122 serum levels correlated with markers for hepatic injury [68,79,83].

### 5.3. miR-133a

miR-133a represents one of the most intensely studied and best characterized miRNAs to date. While it has been initially classified as a myocyte-specific “myomiRNA”, recent studies suggested a role for miR-133a in organ fibrosis, cancer development, and inflammation [84,85]. Alterations in circulating miR-133a concentrations were previously described in manifold diseases such as liver fibrosis, cardiac hypertrophy, diabetes, and various cancers, all representing pathologies associated with a systemic inflammatory response. In line to these findings, Rau *et al.* [10] demonstrated that the induction of Gram-positive bacterial infection in mice was associated with an up-regulation of a panel of nine miRNAs, including mir-133a-1-3p, mir-133a-2-3p, mir-133a-1-5p, and mir-133b-3p. Similarly, we found elevated levels of miR-133a in C57/Bl6 mice after induction of sepsis by using the cecal pole ligation and puncture (CLP) model. Notably, these results could be translated into human sepsis, because concentrations of circulating miR-133a were significantly elevated in a large cohort of critically ill patients when compared to healthy controls. In these patients, elevated miR-133a levels correlated with septic disease and organ dysfunction. High miR-133a levels indicated poor survival and represented an independent predictor of mortality [10]. Thus, miR-133a might represent a promising serum based biomarker in diagnosis and monitoring of septic diseases.

### 5.4. miR-150

miR-150 is part of a group of miRNAs including the miR-17-92 cluster, miR-155, miR-181, and miR-223 with nearly selective expression in immune cells [86]. Overexpression of miR-150 in B-cells resulted in decreased levels of c-Myb and prevented the transition from pro-B to pre-B cells. Consequently, deletion of miR-150 in mice was associated with an expansion of splenic and peritoneal B-cells resulting in increased levels of certain immunoglobulins. On a functional level, down-regulation of miR-150 was described in cell lines as well as in primary leukocytes of human volunteers upon treatment with LPS [87]. Concordantly, miR-150 knockout mice demonstrated significant changes in their responses towards different inflammatory stimuli [26], suggesting a deep integration of miR-150 in the process of immune cell activation during inflammation and sepsis.

miR-150 was among the first miRNAs that were examined in patients with critical illness and sepsis. By applying microarray based gene expression analysis, different authors identified miR-150 as part of panels of miRNAs that were deregulated in leukocytes/PBMC of sepsis-patients compared to healthy controls [58,74,88]. Interestingly, these changes were reflected by concordant alterations of miR-150 serum levels: Vasilescu and colleagues found lower miR-150 concentrations in a cohort of 16 patients with abdominal sepsis [58]. Low miR-150 serum levels correlated with elevated SOFA scores and severity of sepsis. In line, Ma *et al.* [66] reported lower levels of miR-150 in two independent cohorts of patients with sepsis compared to patients with non-infectious SIRS or healthy controls. We recently analyzed levels of circulating miR-150 in a large and well characterized cohort of 223 critically ill patients and 76 healthy controls. Similar to the previous reports, we found lower miR-150 concentrations in patients with septic disease, however differences were small and failed statistical significance [11], highlighting that the potential of miR-150 measurements to differentiate between septic and non septic disease is rather limited. Nonetheless, we observed a strong correlation between low miR-150 levels and an impaired patients’ prognosis in critical illness, suggesting that miR-150 rather has a role as a prognostic rather than diagnostic tool [11]. In line, Vasilescu and colleagues demonstrated that low concentrations of miR-150 were correlated to elevated levels of proinflammatory cytokines [58].

### 5.5. miR-223

miR-223 was initially described as a key modulator of hematopoietic lineage differentiation [89]. Moreover, miRNA expression analyses revealed that miR-223 is deregulated in many inflammation-related disorders. miR-223 is overexpressed in the colonic mucosa and stools of mice and patients with inflammatory bowel diseases (IBD) [90]. Moreover, elevated expression of miR-223 was found in the synovia of patients with rheumatoid arthritis, and silencing of miR-223 was suggested to suppress collagen-induced arthritis in mice [91]. On a functional level, mIR-223 regulates a whole kaleidoscope of genes with a well-established role in the regulation of immune responses, including Artemin, Granzyme B, IGFR1, IKKα, Mef2c, NLRP3, Roquin, STAT3, and Stathmin, suggesting a key role of miR-223 in the regulation of immune responses [89]. In line, miR-223 mutant mice displayed an increased immune response towards infectious agents such as *Candida albicans* and showed elevated tissue destruction in response to LPS. Essandoh *et al.* [92,93] demonstrated that the loss of miR-223 was associated with a more severe inflammation in sepsis and lead to enhanced mortality in miR-233^−/−^ mice compared to wild-type controls, highlighting a potential role of miR-223 in the pathophysiology of sepsis. Wang and colleagues found lower levels of miR-223 in a cohort of 50 patients with sepsis compared to patients with SIRS or healthy controls. Interestingly, miR-223 concentrations were unaffected by non-infectious SIRS, suggesting that miR-223 might serve as a diagnostic tools for distinguishing between infectious and non-infectious SIRS. However, upregulation of miR-223 serum levels were described in another cohort of 166 patients with sepsis compared to healthy controls. Notably, in this respective cohort, elevated miR-223 levels were indicative for the severity of sepsis, which was in line to a recent report from pediatric sepsis patients, which displayed significantly higher levels of miR-146a and miR-223 compared to control children [71]. Again elevated miR-223 positively correlated with higher TNF-α concentrations, disease severity and an impaired patients’ prognosis. We analyzed miR-223 serum concentrations in a large cohort of critically ill patients and failed to detect significant alterations in serum miR-223 concentrations between non-infectious critical illness and sepsis. The conflicting results between all of these studies might be multifactorial: on one hand, very different cohorts of patients were analyzed. Hence, it cannot be excluded that a yet unknown factor influencing miR-223 concentrations might have biased some of the published results. On the other hand, experimental procedures significantly varied between the studies: while we used spiked-in RNA (SV40) for normalization of miR-223 serum levels, other authors normalized their data by using internal reference genes (references see above). Of note, besides sepsis, conflicting results for miR-223 serum levels have also been described in patients with HCC or chronic hepatitis B, highlighting the need for further efforts in standardization in experimental procedures.

### 5.6. miR-297 and miR-574-5p

Wang *et al.* [60] identified miR-297 and miR-574-5p as potential prognostic biomarkers in critically ill/septic patients by using genome-wide scans on miRNA levels in samples from 12 patients that survived sepsis and 12 matched patients that succumbed to death during septic disease. Notably, these array based data could be confirmed in a collective of 118 critically ill patients, in which alterations in miR-574-5p levels could be correlated to an unfavorable prognosis. Notably, by combining sepsis stage, Sepsis-Related Organ Failure Assessment (SOFA) score, and levels of miR-574-5p in multivariable logistic regression analyses, the predictive power of miR-574-5p measurements for patients’ prognosis could be further increased, suggesting miRNAs serum levels might become part of complex clinical prognosis scores to guide clinical decisions in the setting of medical intensive care unit (ICU) treatment.

### 5.7. miR-4772

Ma and colleagues applied the method of massively parallel sequencing of microRNAs for screening of deregulated miRNA candidates on pooled RNAs of four blood samples from healthy volunteers, SIRS and sepsis patients [66]. Results were validated using quantitative real-time PCR in 23 septic patients, 22 SIRS patients and 21 healthy controls as well as in another, independent validation cohort. These analyses revealed significantly higher concentrations of circulating miR-150 and of members of the miR-4772 family in patients with sepsis compared to healthy controls. However, levels of these miRNAs were not significantly different between the sepsis and SIRS groups. On a cellular level, miR-4772 was upregulated in primary peripheral blood monocytes after treatment with LPS, providing a mechanism driving the upregulation of miR-4772 levels in sepsis [66].

### 5.8. Other miRNAs

A comprehensive overview on other miRNA deregulated in the blood of patients with sepsis in provided in Table 1.

## 6. Conclusions

In the last years, miRNAs have been suggested as biomarkers in the context of sepsis. However, several problems needs to be solved before a use in clinical routine can be further considered: there are striking inter-study variances of miRNA-regulation patterns in the different cohorts of patients with sepsis, which are most likely due to a lack in standardization of sample collection, data normalization, and analysis. As an example, despite years of research there is still no consensus on the optimal normalization strategy for miRNA analysis from serum. Many authors suggest the use of internal genes, such as miR-16 or snU6, for normalization. However, we [94] have recently shown that snU6 might be differentially regulated between healthy controls and critically ill/sepsis patients, highlighting that this strategy might bias such analysis. Based on such data it was suggested that using spiked-in RNAs such as SV40 will provide higher accuracy in this setting. However, if these problems can be solved, miRNAs offer attractive options as “next generation” biomarkers in the context of critical illness and sepsis. Additionally, simply indicating the presence of bacterial infection and sepsis, it was recently suggested that circulating miRNA could also be indicative for more specific features of critical illness and sepsis: Wu *et al.* [95] showed that serum levels of seven miRNAs namely miR-133a, miR-133b, miR-122, miR-205, miR-1899, miR-714, and miR-291b are specifically regulated in the case of Gram-positive infection with *Staphylococcus aureus*, while levels of miR-16, miR-17, miR-20a, miR-26a, miR-26b, miR-106a, miR-106b, and miR-451 were selectively higher in Gram-negative sepsis with *E. coli*. Other specific changes in miRNA expressions could be found in various bacteria such as *Helicobacter pylori* [96], *Listeria monocytogenes* [97], *Mycobacterium tuberculosis* [98], *Salmonella enterica* [99], *Staphylococcus aureus* [100], *Brucella melitensis* [101], and *Pseudomonas aeruginosa* [102,103] or parasitic infection with *Cryptosporidium parvum* [104,105,106], *Leishmania* [107], and *Plasmodium vivax* [108]. Thus, analysis of circulating miRNA bear the potential to serve as point of care diagnostic tests allowing rapid initiation of directed treatment by circumventing time intensive microbiological confirmation of sepsis and infection.
